# Fatty Kidney Disease: From Renal Lipid Dysregulation to Fibrosis

**DOI:** 10.3390/biology15131021

**Published:** 2026-06-26

**Authors:** Toshiharu Onodera, Naoki Morimoto, Yosuke Okuno, Iichiro Shimomura

**Affiliations:** Department of Metabolic Medicine, Graduate School of Medicine, The University of Osaka, Yamadaoka, Suita 565-0871, Osaka, Japan

**Keywords:** fatty kidney disease, renal fibrosis, fatty acid oxidation

## Abstract

Progression to fibrosis is a major complication of chronic kidney disease (CKD) in obesity, type 2 diabetes, hypertension, and metabolic syndrome, yet effective antifibrotic therapies remain limited. Here, we review how disordered renal energy metabolism—ectopic lipid accumulation, impaired fatty acid oxidation (FAO), and a compensatory shift toward glycolysis—drives tubulointerstitial fibrosis in fatty kidney disease. Lipid overload in tubular, glomerular, and vascular cells arises from increased uptake via scavenger and lipoprotein receptors, enhanced lipogenesis, and reduced lipid catabolism and clearance. Spatial lipidomic studies further reveal nephron-segment-specific lipid signatures and obesity-associated oxidized phospholipids linked to glomerular inflammation. Lipotoxicity, mitochondrial damage, and associated innate-immune signaling, ferroptosis, cellular senescence, and adipose-derived mediators (including leptin, adiponectin, and a locally active renin–angiotensin system) converge on myofibroblast activation from pericytes, fibroblasts, and other resident cells. We discuss established and emerging therapies targeting this metabolic axis—peroxisome proliferator-activated receptor-α (PPARα) modulators, sodium–glucose cotransporter-2 (SGLT2) inhibitors, glucagon-like peptide-1 (GLP-1) receptor agonists, and the mineralocorticoid receptor antagonist finerenone—and propose that restoring metabolic flexibility, by rescuing FAO while limiting maladaptive glycolysis, offers a promising disease-modifying strategy for fatty kidney disease.

## Simple Summary

Kidney disease is becoming more common worldwide, driven largely by rising rates of obesity and type 2 diabetes. The kidney is one of the most energy-demanding organs in the body, and its cells rely on a careful balance between burning fat and burning sugar to function properly. When this balance is disturbed—as it is in obesity and diabetes—fat begins to accumulate inside and outside kidney cells, the small structures inside cells that produce energy, called mitochondria, stop working efficiently, and the kidney loses its capacity to produce energy. Over time, this energy failure triggers inflammation and replaces healthy kidney tissue with scar tissue, a process that ultimately leads to kidney failure. This review brings together current knowledge of how the healthy kidney generates and uses energy, how that machinery breaks down in obesity, diabetes, and other forms of metabolic stress, and how this metabolic breakdown drives irreversible kidney scarring. We also discuss how the fat tissue surrounding the kidney contributes to injury, and we review emerging treatments designed to restore normal kidney energy use. A better understanding of these pathways may help doctors detect kidney damage earlier and develop new therapies to slow or prevent kidney failure.

## 1. Introduction

As the global prevalence of obesity continues to rise, so does the incidence of type 2 diabetes mellitus. Diabetic nephropathy, one of the most clinically important diabetic complications, typically presents with persistent albuminuria; together with chronic hyperglycemia, sustained albuminuria progressively damages the kidney and ultimately leads to end-stage renal disease (ESRD). During this progression, renal fibrosis develops through excessive collagen deposition in the extracellular matrix (ECM), accompanied by chronic inflammation. A variety of insults—persistent proteinuria, uncontrolled hypertension, metabolic stress, obesity, and chronic hypoxia—converge to drive this fibrotic process. Because these risk factors tend to cluster in the same individual, they are collectively referred to as metabolic syndrome (historically termed the “deadly quartet”), which is characterized by visceral fat accumulation together with dyslipidemia, hypertension, and type 2 diabetes.

Obesity itself constitutes an independent risk factor for ESRD [1]. In particular, the chronic excess of circulating lipids that accompanies obesity culminates in ectopic fat deposition in the kidney, a hallmark of obesity-related renal disease [2]. Recent advances in imaging mass spectrometry have further revealed that the diabetic kidney exhibits pronounced alterations in lipid distribution in both murine and human tissues [3]. In diabetic kidneys, lipid-uptake genes—including *CD36*, *SR-A1*, *LOX-1*, and *LDLR*—are highly induced, whereas fatty-acid-oxidation genes, such as *ACOX1* and *CPT1*, are markedly suppressed [4]. More recent studies have uncovered additional layers of renal lipid dysregulation: aberrant lipid synthesis through the SREBP-1/2 and SOAT1 (historically labeled “ACAT1” in the cholesterol literature, but distinct from HGNC *ACAT1*) axes [5], impaired lipid degradation [6], and endothelial VEGF-B signaling [7] each amplify renal lipid accumulation and the ensuing cytotoxicity.

Although the precise spatial pattern of renal lipid accumulation remains incompletely defined, ectopic lipid deposition affects multiple kidney cell types, including proximal tubular cells [8], podocytes [9], mesangial cells [10], and endothelial cells [7].

In this review, we integrate genetic, spatial, and cellular evidence to clarify how disordered renal lipid handling and mitochondrial–glycolytic rewiring drive fibrosis in fatty kidney disease, with attention to adipose tissue–derived signals that further amplify renal injury. We then survey emerging therapeutic strategies—including PPARα modulators, SGLT2 and GLP-1 receptor agonists, and mineralocorticoid receptor antagonists—aimed at restoring renal metabolic flexibility in obesity- and diabetes-associated kidney disease.

### 1.1. The Concept of Fatty Kidney Disease

Although no unified definition yet exists, fatty kidney disease has re-emerged over the past decade or so as an increasingly recognized clinical entity within CKD, driven by the obesity pandemic and by rising renal-biopsy detection of obesity-related glomerulopathy (ORG) [11,12]. Paralleling the reframing of fatty liver disease as MASLD, nephrology has proposed metabolic dysfunction–associated kidney disease (MDAKD) as an umbrella term for CKD arising in the context of metabolic dysfunction—obesity, insulin resistance, diabetes, dyslipidemia, and hypertension—encompassing diabetic kidney disease and obesity-related kidney disease [13].

Within this landscape, fatty kidney disease and ORG are best understood as concepts defined along different axes. Fatty kidney disease is an organ-level, imaging-defined concept centered specifically on ectopic lipid accumulation in and around the kidney, quantified as renal fat fraction by MRI/CT; one proposed operational definition sets fatty kidney at a renal fat fraction ≥4% by MRI [14]. Rather than a discrete subcategory of MDAKD, it represents a mechanistic dimension that cuts across the MDAKD spectrum. ORG, by contrast, is a histologically defined entity: in the setting of obesity, and after exclusion of diabetic nephropathy and hypertensive nephrosclerosis, it is characterized by glomerulomegaly with or without (perihilar) focal segmental glomerulosclerosis [12].

Two broad mechanisms link renal and perirenal fat to kidney injury. (1) Ectopic lipid deposition within the renal parenchyma produces lipotoxic injury to podocytes, tubular, and mesangial cells and contributes to acute kidney injury and CKD, including diabetic nephropathy, ORG, age-related kidney disease, and polycystic kidney disease [4,15]. (2) Accumulation as renal sinus and perirenal fat mechanically compresses the kidney, impairs renal hemodynamics, and promotes inflammation, thereby compromising renal function [16,17].

In DKD, renal parenchymal fat is associated with CKD and increases across disease stages [18,19], and lipotoxicity-mediated tubular injury has been proposed as a primary event preceding glomerular dysfunction [15]. The close association of perirenal and renal sinus fat with increased CKD risk is well established [16,20], and perirenal adipose tissue (PRAT) has been reported to carry superior predictive value for DKD progression compared with systemic/general adiposity measures such as BMI and total, subcutaneous, or visceral fat [19,20].

With respect to hypertensive nephrosclerosis, renal sinus fat excess (sinus lipomatosis) compresses the low-pressure veins and lymphatics within the renal sinus and raises intrarenal hydrostatic pressure. In obese rabbits, kidneys were ~20% larger because of renal lymphatic compression, despite no detectable parenchymal fat accumulation [17]. Clinically, greater renal sinus fat is associated with hypertension and GFR decline independently of other fat compartments [21,22] (PMID: 20837881; 38555192). Mechanistically, renal sinus fat raises intra-abdominal pressure and compresses low-pressure renal venous structures, leading to renal volume expansion, increased renal interstitial pressure, and RAAS activation, which in turn contributes to hypertension, atherosclerosis, and insulin resistance [17].

### 1.2. Pathogenesis of Fatty Kidney Disease and Implications for Fibrosis

Building on this concept, it is worth clarifying how renal lipid dysregulation relates to the classical drivers of kidney injury. We position ectopic lipid accumulation primarily as a convergent amplifier that is engaged by—and in turn intensifies—hemodynamic and glycemic insults, rather than as a replacement for hypertension and hyperglycemia as the principal causes of functional decline. In defined settings, however, such as obesity and metabolic syndrome without overt diabetes, lipid overload can also act as a partially independent, initiating driver, with lipotoxic tubular injury preceding glomerular dysfunction. These roles are not mutually exclusive: both converge on shared effectors—mitochondrial dysfunction, oxidative stress, and pro-fibrotic signaling—that drive tubulointerstitial fibrosis.

Consistent with this view, hypertension and chronic hyperglycemia—rather than renal lipid accumulation per se—remain the major drivers of declining kidney function: chronic hypertension causes nephrosclerosis, whereas diabetes leads to diabetic nephropathy. Nevertheless, ectopic renal lipid accumulation is increasingly recognized as a pathogenic amplifier of these processes, arising from an imbalance between lipid influx and lipid catabolism in the kidney.

Lipid influx is augmented by a range of metabolic stresses, including hyperglycemia, hyperlipidemia, and excessive fructose intake [23,24]; fructose, in particular, induces intrarenal lipid accumulation via ChREBPβ-mediated lipogenic transcription [25]. Conversely, impaired lipid consumption—most notably through reduced fatty-acid oxidation (FAO)—exacerbates renal lipid deposition. Kang et al. demonstrated that the expression of genes involved in FAO, including CPT1A, ACOX1, PPARA, and PPARGC1A, was reduced in human CKD kidneys and mouse models of renal fibrosis, accompanied by lipid accumulation in tubular epithelial cells. Furthermore, tubular epithelial cell–specific overexpression of PPARGC1A (Pax8rtTA/TRE-PPARGC1A mice) and treatment with the PPARα agonist fenofibrate restored FAO-related gene expression and attenuated renal fibrosis in unilateral ureteral obstruction and folic acid nephropathy mice models. In contrast, CD36 overexpression (Pax8rtTA/TRE-CD36 mice) induced lipid accumulation but was insufficient to cause substantial renal fibrosis. Collectively, these findings suggest that impaired FAO, rather than lipid accumulation per se, plays a critical role in the progression of tubular injury and tubulointerstitial fibrosis [26]. Consistently, restoring tubular *Cpt1a* expression protects from kidney fibrosis by improving mitochondrial homeostasis [27], whereas genetic loss of *Ppara* aggravates lipid deposition and accelerates fibrosis in multiple injury models [28,29,30]. More recently, impaired carnitine-dependent FAO—mediated through organic cation/carnitine transporter 2 (OCTN2) and the downstream mitochondrial carnitine shuttle—has been demonstrated in experimental and human diabetic kidney disease, where it promotes renal lipid accumulation [31], underscoring carnitine-dependent FAO as a critical safeguard against renal lipotoxicity.

### 1.3. Genetic Determinants and Molecular Basis of Fatty Kidney Disease

**Monogenic causes.** Three single-gene defects show that disordered renal lipid handling alone can drive kidney injury. Biallelic loss-of-function variants in *SLC22A5* (OCTN2) cause primary systemic carnitine deficiency, characterized by defective tubular carnitine reabsorption and multi-organ steatosis [32,33]; more recently, impaired carnitine-dependent FAO has been demonstrated in experimental and human diabetic kidney disease, extending the relevance of OCTN2 biology beyond Mendelian carnitine deficiency [31]. *PPARA* encodes the master regulator of proximal-tubule β-oxidation, and reduced renal PPARα expression in fibrotic human kidney correlates with CKD progression [26,34], with clinical meta-analysis supporting renoprotective effects of PPARα-activating fibrates [35]; *Ppara*-deficient mice show tubular lipid accumulation and exaggerated fibrosis after ischemic, septic, or proteinuric injury, which is reversed by pharmacological PPARα activation [28,29,30]. *SOAT1* esterifies free cholesterol; SOAT1 dysregulation drives free-cholesterol accumulation in podocytes, mitochondrial dysfunction, and proteinuria in obesity- and diabetes-associated kidney disease, although no Mendelian *SOAT1* syndrome has been described [36,37] (Table 1).

**Table 1:** Genetic and molecular landscape of fatty kidney disease.

Category	Gene	Association with Fatty Kidney	
Monogenic	SLC22A5 (OCTN2)	Primary systemic carnitine deficiency with tubular carnitine wasting and multi-organ steatosis; impaired carnitine-FAO is also seen in human/experimental DKD	Human [31,32,33]; Mouse [31]
Expression-driven	PPARA	Master regulator of tubular β-oxidation; reduced renal expression in fibrotic kidney correlates with CKD; PPARα-activating fibrates are renoprotective in meta-analysis. No Mendelian disease	Human [26,34]; Mouse [28,29,30]
Expression-driven	SOAT1 (≠ *HGNC ACAT1*)	Cholesterol esterification; dysregulation drives free-cholesterol accumulation in podocytes → mitochondrial dysfunction → proteinuria in obesity/DKD	Human [37]; Mouse [36,37]
SNPs/polygenic	PPARG	Pro12Ala (rs1801282) Ala allele ↓ DN risk (meta-OR ≈ 0.74); tubular Pparg maintains renal metabolic heterogeneity	Human [38,39]; Mouse [40]
Expression-driven	CD36, OLR1 (LOX-1), EGR1	Renal expression up-regulated in CKD/DKD biopsies with dyslipidemia; Cd36 KO restores FAO and attenuates lipotoxicity. Robust human variant evidence only for CD36	Human [34]; Mouse [41]
Emerging (GWAS → mechanism)	ACSS2	Risk variants act as eQTLs lowering ACSS2 expression → protect from CKD; deletion/inhibition reduces DNL, NADPH/ROS, NLRP3 pyroptosis, and fibrosis	Human [42]; Mouse [42,43,44]
Emerging regulator	CCDC92	Cardiometabolic-GWAS-derived; deficiency alleviates podocyte -specific lipotoxicity (not tubular) via PA28α/ABCA1 cholesterol-efflux axis in DKD	Human [45,46]; Mouse [45,46]
Lipid mediator/heme-binding	mPGES2 (PTGES2)	Heme-binding (not canonical PGE2 synthase); sequesters heme from Rev-Erbα → derepresses FABP5 → renal lipid uptake. Pharmacologic blockade attenuates DKD	Human [47]; Mouse [47,48]
Autophagy/lysosomal program	TFEB, autophagy-lysosome	Reduced renal autophagy → progressive tubular and podocyte injury in DKD; lysosomal restoration counters lipotoxicity; podocyte autophagy is essential in aging	Human [49,50,51]; Mouse [50,52]
Mitochondrial biogenesis/NAD^+^	PGC-1α (PPARGC1A) NAD^+^	PGC-1α–driven mitochondrial biogenesis and NAD^+^ metabolism rescue tubular FAO and energy balance; tubular NAD^+^ activation prevents CKD progression	Human [34]; Mouse [26,53,54]

→, leads to; ↓, decreased. Abbreviations: CKD, chronic kidney disease; DKD, diabetic kidney disease; DN/DKD, dia-betic nephropathy; DNL, de novo lipogenesis; eQTL, expression quantitative trait locus; GWAS, genome-wide associa-tion study.

**Common variants and polygenic susceptibility.** The *PPARG* Pro12Ala (rs1801282) Ala allele is associated with reduced diabetic nephropathy risk by meta-analysis (OR ≈ 0.74) [38,39], and tubular *Pparg* signaling maintains renal metabolic heterogeneity and protects against tubular injury in mice [40]. In contrast, evidence linking the lipid-handling genes *CD36*, *OLR1* (LOX-1), and *EGR1* to fatty kidney disease rests primarily on transcriptional rather than human genetic data: although robust polymorphism–CKD associations have not been established (emerging support exists only for *CD36* variants), their renal transcript and protein levels are consistently up-regulated in CKD/DKD biopsies from dyslipidemic patients [34], and *Cd36*-knockout or pharmacological blockade attenuates tubular and podocyte lipotoxicity by restoring FAO and mitochondrial function in diabetic rodents [41].

**Emerging GWAS-to-mechanism loci.** Common variants at *ACSS2*, encoding the cytosolic acetyl-CoA synthetase driving de novo lipogenesis (DNL), act as eQTLs lowering ACSS2 expression and protecting from CKD; *ACSS2* deletion or pharmacological inhibition reduces NADPH depletion, ROS, NLRP3-mediated pyroptosis, histone crotonylation, and fibrosis in multiple mouse models [42,43,44]. *CCDC92*, identified through cardiometabolic GWAS, drives podocyte (rather than tubular) lipotoxicity in diabetic kidney disease through the PA28α/ABCA1 cholesterol-efflux axis [45,46]. Microsomal prostaglandin E synthase-2 (*mPGES2*/*PTGES2*) has been reframed as a heme-binding rather than canonical PGE2-synthesizing enzyme: it sequesters heme from Rev-Erbα to derepress FABP5 and promote lipid uptake, and genetic or pharmacological mPGES2 blockade attenuates renal lipotoxicity in diabetic mice [47,48].

**Protective autophagic and mitochondrial programs.** Autophagic-lysosomal flux and mitochondrial biogenesis cooperate to restrain renal lipotoxicity. Reduced renal autophagy is associated with progressive tubular and podocyte injury in diabetic and inflammatory kidney disease [49,50,51], and podocyte autophagy is essential for glomerular homeostasis in aging mice [52]. Tubular lysosomal restoration counters lipotoxic podocyte injury in diabetic kidney disease [50], while PGC-1α–driven mitochondrial biogenesis and NAD^+^ metabolism rescue tubular FAO, energy balance, and structural recovery in ischemic, septic, and lipotoxic injury [26,53], and renal tubular NAD^+^ activation prevents CKD progression [54]. Several other proteins reinforce this protective network: AMPK with ULK1 helps kidney cells digest stored fat, NRF2 limits oxidative damage, FOXO3 drives autophagy and antioxidant genes, and SIRT3 sustains mitochondrial fat burning.

Together, these loci converge on five interlocking nodes—fatty-acid uptake (*SLC22A5*, *CD36*, *OLR1*), β-oxidation (*PPARA*, *PGC-1α*), de novo lipogenesis (*ACSS2*), cholesterol esterification and efflux (*SOAT1*, *CCDC92*), and autophagic/mitochondrial quality control (*TFEB*, AMPK, NRF2)—defining the genetic architecture of fatty kidney disease and delineating therapeutically tractable points of intervention [34].

## 2. Mechanisms Linking Lipid Dysmetabolism to Renal Fibrosis

### 2.1. Mechanisms by Which Lipid Accumulation Drives Renal Fibrosis

As is well recognized in hepatic steatosis, impaired fatty acid metabolism and lipid accumulation are also important contributors to fibrosis in the kidney. PCSK-9 overexpression-mediated dyslipidemia caused renal fat deposition accompanied by renal fibrosis, with increased ferroptosis and ER stress [55]. Deficiency of acyl-CoA thioesterase 12 (Acot12), an enzyme that hydrolyzes acyl-CoA thioesters to free fatty acids (FA) [56], exacerbates renal fibrosis independently of PPARα and is accompanied by reduced autophagic degradation of peroxisomes (pexophagy). Observations in the lung help to clarify the mechanisms of lipid-mediated fibrosis. In idiopathic pulmonary fibrosis (IPF), altered TG and cholesterol metabolism [57], together with bioactive lipids, such as PGE2, ceramides, and sphingomyelins, promote myofibroblast activation and extracellular matrix deposition via TGFβ [58]. Although changes in lipid-metabolism-related genes occur across many IPF cell types, they are most pronounced in macrophages [59]. The prevalence of metabolic dysfunction-associated steatotic liver disease (MASLD) is on the rise. In MASLD, progressive lipid accumulation drives lipotoxicity, which in turn triggers ER stress, oxidative stress, and ferroptosis. Lipotoxicity also up-regulates Fas and FasL, recruiting caspase-8 to induce apoptosis [60]. Although the kidney, lung, and liver differ substantially in cellular composition and physiological function, these observations suggest that lipotoxicity-induced ER stress, oxidative stress, and ferroptosis may represent conserved profibrotic pathways across organs.

### 2.2. Spatial Distribution of Renal Lipid Species in Health and Disease

Renal lipid metabolism is highly compartmentalized, matching each segment’s metabolic demand: cortical proximal tubules depend on fatty-acid oxidation (FAO) to power their high reabsorptive load [61,62], whereas medullary segments rely more on glycolysis under chronic hypoxia [63,64] (Table 2). A human spatial lipid atlas shows that each nephron segment carries a distinct lipid signature [65]: sphingomyelins and GA1 gangliosides in the glomerulus; long-chain sphingomyelins and polyunsaturated phosphatidylethanolamines in the proximal tubule, with an apical-to-basolateral sphingomyelin gradient [66]; α-hydroxylated sulfatides in the thick ascending limb [67,68]; lysophospholipids in the distal tubule; and structural phospholipids forming “leak-proof but flexible” membranes in the collecting duct [65,68]. The healthy lipid map is thus a chemical readout of metabolic specialization, and it is already reshaped by adiposity—in individuals with BMI > 35, oxidized phosphatidylcholines (PAz-PC) accumulate in the glomerulus and are linked to inflammation and incipient glomerular disease [65].

Disease rewrites this map. In diabetic kidney disease (DKD), the shift is consistently away from oxidation and toward lipogenic storage: in diabetic mouse glomeruli, SM (d18:1/16:0) accumulates and enhances mesangial-cell glycolysis [69], alongside increases in gangliosides, sulfatides, lysophospholipids, and PE that implicate oxidative-stress pathways in early disease [70]. In long-standing human DKD, spatial metabolomics combined with single-cell RNA sequencing (KPMP cohort) shows injured thick ascending limb and proximal tubule cells adopting a lipogenic phenotype—increased lipid synthesis, suppressed β-oxidation, and triglyceride, glycerophospholipid, and sphingolipid enrichment in the inner medulla [71]—so the very segments that define the healthy FAO-based map invert their program. Mirroring these tissue-level changes at the systemic level, circulating ceramide profiles may provide a clinically accessible readout of renal lipotoxicity. In a prospective cohort of 1746 Southeast Asian patients with type 2 diabetes followed for a median of 7.7 years, higher Cer16:0/Cer24:0, Cer18:0/Cer24:0, and Cer24:1/Cer24:0 ratios independently predicted rapid kidney function decline and progression to end-stage kidney disease [72]. Obesity-related kidney disease shows a partly overlapping signature—glomerular SM (d18:1/16:0) and PAz-PC gain with loss of structural phospholipids [65]—defining a shared “metabolic fatty-kidney” fingerprint that links systemic metabolic stress to a stereotyped renal lipid response.

Other injuries remodel renal lipids through distinct, largely non-overlapping signatures, underscoring the specificity of the metabolic pattern: ceramide accumulation with sphingomyelin and phosphatidylcholine loss in aging [73]; ether-lipid and membrane turnover in ischemic AKI [74], progressing to lysocardiolipin and lysophospholipid accumulation in severely ischemic transplant kidneys [75]; a plasma oxylipin fingerprint in sepsis-associated AKI [76]; S3-segment phospholipid and sulfatide redistribution with cardiolipin loss in cisplatin nephrotoxicity [77,78]; and storage-type glycosphingolipid signatures in genetic and inflammatory disease—sulfatides in Alport syndrome [79], hexosylceramides and lactosylceramides in lupus nephritis [80], and Gb3/Ga2Cer in the Fabry kidney [81] (Table 2).

**Table 2:** Spatial distribution of renal lipid species in physiological and pathological conditions.

Condition	Kidney Region/Cell Type	Key Lipid Species (Change)	Species	Ref
Physiological lipid distribution (healthy kidney)				
Healthy	Glomerulus (GL)	SM (18:1; 2O/16:0); gangliosides (GA1 species)	Human	[65]
Healthy	Proximal tubule (PT)	Long-chain sphingomyelins (C18–C26); PS (38:4); PE (38:4) polyunsaturated	Human	[65]
Healthy	Proximal tubule (PT) (apical > basolateral gradient)	SM d34:1, SM d34:2 enriched on luminal/apical membrane	Human	[66]
Healthy	Thick ascending limb (TAL)/medulla	Sulfatides (SHexCer); AS-SM4s with C22/C24 α-hydroxy fatty-acyl chains	Mouse	[67,68]
Healthy	Distal tubule (DT)	Lysophospholipids: LPC (16:0), LPE (18:0)	Human	[65]
Healthy	Collecting duct (CD)	PC (32:1), PC (35:1) [exclusive markers]; PE (36:1), PE (38:1); SM (40:0;2O), SM (42:0;3O); SHexCer (t18:0/h24:0)	Human/Mouse	[65,68]
Pathological alterations				
Obesity (BMI > 35)	Glomerulus	↑ Oxidized phosphatidylcholine (PAz-PC)—linked to chronic inflammation and glomerular disease	Human	[65]
Diabetic kidney disease	Glomerulus (mesangial cells)	↑ SM (d18:1/16:0)—promotes glycolysis and ↑ ATP/AMP ratio	Mouse	[69]
Diabetic kidney disease	Glomerulus + tubules	↑ GM3 gangliosides, sulfatides, LPC, LPE, PE—glucose-independent oxidative stress	Mouse	[70]
Obesity-related kidney disease	Glomerulus	↑ SM (d18:1/16:0); ↑ oxidized PC (PAz-PC); ↓ structural phospholipids (LPE, PC, PS)—glomerulosclerosis-associated membrane remodeling	Human	[65]
Aging kidney	Cortex + medulla	↑ Ceramides (e.g., Cer d18:1/14:0, 20:0)—pro-apoptotic, TGF-β signaling; ↓ SM, ↓ PC	Mouse	[73]
Acute kidney injury (AKI)	Proximal tubule	↑ Plasmanyl PC (PC O-38:1); transient ↑ then ↓ plasmalogen PE (PE O-42:3)—antioxidant lipid consumption	Mouse	[74]
Severe renal ischemia	Whole tissue (transplant kidneys)	↑ Lysocardiolipins, LPC, LPI—mitochondrial dysfunction and membrane breakdown	Human	[75]
Cisplatin nephrotoxicity	Proximal tubule S3 segment	Redistribution of PC, PE, PI, sulfatides; ↑ lysophospholipids; ↑ PI (38:4); ↓ cardiolipin; ↑ cholesteryl esters	Mouse/Rat	[77,78]
Alport syndrome	Renal tubules	↑ Sulfatides (SHexCer)	Mouse	[79]
Lupus nephritis	Glomerulus + urine	↑ Hexosylceramides (HexCer); ↑ lactosylceramides (LacCer); early urinary LacCer elevation as a biomarker	Mouse/Human	[80]
Fabry disease	Whole kidney (α-Gal A-KO)	Widespread ↑ globotriaosylceramide (Gb3); ↑ galabiosylceramide (Ga2Cer)	Mouse	[81]
Long-standing DKD (>10 y)	Injured TAL (iTAL); injured PT (iPT); inner medulla	↑ Triglycerides, glycerophospholipids, sphingolipids in inner medulla; iTAL/iPT cells show increased lipid biosynthesis and decreased β-oxidation (KPMP scRNA-seq + spatial multi-omics)	Human	[71]
T2DM → ESKD	Plasma biomarker (*n* = 1746; 7.7-y follow-up)	Plasma ceramide ratios—Cer 16:0/Cer 24:0, Cer 18:0/Cer 24:0, Cer 24:1/Cer 24:0—predict rapid kidney function decline; Cer 16:0/Cer 24:0 and Cer 24:1/Cer 24:0 also predict ESKD	Human	[72]
Sepsis-associated AKI	Plasma (*n* = 67 SA-AKI vs. 20 controls)	Differential oxylipins: 5(S),12(S)-DiHETE, 5-isoPGF2VI, 5,6-DiHETrE, 11,12-EET, 9,10-DiHOME—discriminate SA-AKI stages	Human	[76]

Legend: Yellow = Human; Green = Mouse/Rat; Pink = Both human and mouse evidence; GL = glomerulus, PT = proximal tubule, TAL = thick ascending limb, DT = distal tubule, CD = collecting duct. Symbols: ↑, increased; ↓, decreased; →, leads to.

Across these conditions, lipid remodeling converges on three axes—deranged fatty-acid and mitochondrial membrane metabolism, oxidative membrane damage, and ceramide- and glycosphingolipid-driven pro-fibrotic signaling—descriptive signatures that mass-spectrometry imaging and single-cell lipidomics are poised to convert into diagnostic biomarkers and therapeutic targets for fatty kidney disease.

### 2.3. Mitochondrial Dysfunction and Metabolic Reprogramming

Mitochondrial dysfunction plays a central role in the pathogenesis of renal fibrosis, the common final pathway of chronic kidney disease (CKD). Under physiological conditions, renal tubular epithelial cells rely predominantly on fatty acid oxidation (FAO) and mitochondrial oxidative phosphorylation (OXPHOS) to meet their high ATP demand. During fibrogenesis, however, tubular cells undergo profound metabolic reprogramming characterized by suppression of FAO, impaired mitochondrial bioenergetics, and a shift toward glycolysis [82,83].

Defective FAO has emerged as a key driver of renal fibrosis. Seminal studies by Kang et al. demonstrated that impaired FAO in tubular epithelial cells induces ATP depletion, dedifferentiation, lipid accumulation, and cell death, thereby promoting fibrotic progression. Transforming growth factor-β1 (TGF-β1) suppresses FAO through SMAD3-mediated inhibition of PPARGC1A signaling [26]. Consistent with this concept, reduced expression of carnitine palmitoyltransferase 1 (CPT1) and PPARα has been associated with lipid accumulation and kidney injury, whereas tubular overexpression of CPT1A restores mitochondrial oxidative metabolism, increases ATP production, and attenuates fibrosis [27]. Peroxisomal FAO may also contribute to renal lipid homeostasis and fibrotic remodeling. ACOX1, the rate-limiting enzyme of peroxisomal β-oxidation, is reduced in fibrotic renal allografts, and ACOX1 deficiency promotes lipid accumulation, extracellular matrix remodeling, and fibrosis through PUFA depletion and ER stress activation [84]. These findings suggest that restoration of FAO represents a promising therapeutic strategy for CKD-associated fibrosis.

Alterations in mitochondrial dynamics also contribute to fibrogenesis. Increased mitochondrial fission mediated by dynamin-related protein 1 (DRP1) promotes mitochondrial fragmentation, mitochondrial reactive oxygen species (mtROS) generation, fibroblast activation, and proliferation. TGF-β1-induced mitochondrial dysfunction and glycolytic reprogramming are partially reversed by DRP1 inhibition, indicating a mechanistic link between mitochondrial dynamics and metabolic remodeling [85]. Conversely, impaired mitochondrial fusion, particularly through reduced mitofusin 2 (MFN2), may disrupt mitochondrial morphology, endoplasmic reticulum–mitochondria contact sites, calcium homeostasis, and bioenergetic function, thereby aggravating podocyte injury, ischemia–reperfusion injury, and fibrotic remodeling [86,87].

In parallel, mitochondrial injury results in the release of mitochondrial DNA (mtDNA) into the cytosol, activating the cGAS–STING pathway and triggering inflammation and fibrosis [88]. Mitochondrial transcription factor A (TFAM), a key regulator of mtDNA replication, transcription, and maintenance, is therefore increasingly recognized as an important determinant of mitochondrial integrity in kidney disease. Loss or suppression of TFAM impairs mtDNA replication, promotes mitochondrial depletion and dysfunction, and may facilitate mtDNA-mediated inflammatory signaling [89]. In diabetic kidney disease, AKAP1–PKC–Larp1-dependent suppression of TFAM has been implicated in podocyte mitochondrial dysfunction [90], whereas SIRT3-dependent regulation of TFAM may promote mitochondrial quality control and protect against acute kidney injury [91].

Sirtuin 3 (SIRT3), a mitochondrial NAD^+^-dependent deacetylase, further links mitochondrial metabolism to renal fibrosis. SIRT3 regulates FAO, OXPHOS, antioxidant defense, and mitochondrial dynamics through deacetylation of key mitochondrial proteins. In diabetic kidneys, SIRT3 deficiency promotes mitochondrial protein hyperacetylation, TGF-β/SMAD activation, hypoxia-inducible factor-1α (HIF-1α) accumulation, PKM2 dimerization, abnormal glycolysis, and fibrotic programming [92]. Thus, SIRT3 loss may couple impaired mitochondrial metabolism to glycolytic reprogramming and profibrotic signaling, whereas restoration of SIRT3 activity may represent a potential therapeutic strategy.

Another hallmark of renal fibrosis is the metabolic shift from OXPHOS toward aerobic glycolysis. Fibrotic kidneys and TGF-β1-treated fibroblasts showed upregulation of glycolytic enzymes, increased glucose uptake, and increased lactate production, demonstrating that aerobic glycolysis is a key feature of renal fibroblast activation. This metabolic shift from oxidative phosphorylation to aerobic glycolysis is associated with myofibroblast activation, and inhibition of aerobic glycolysis, particularly PKM2, has been shown to suppress renal fibrosis [93]. During the transition from acute kidney injury (AKI) to CKD, persistent suppression of FAO and enhanced glycolysis are driven in part by HIF-1α and TGF-β signaling [94,95]. In AKI, increased lactate promotes degradation of Prohibitin 2 (PHB2) through lactylation of ALDH2, thereby impairing mitophagy and inducing mitochondrial structural damage [96]. This metabolic switch limits pyruvate entry into the tricarboxylic acid cycle and favors lactate production, thereby exacerbating mitochondrial dysfunction and fibrosis.

**Figure 1:**
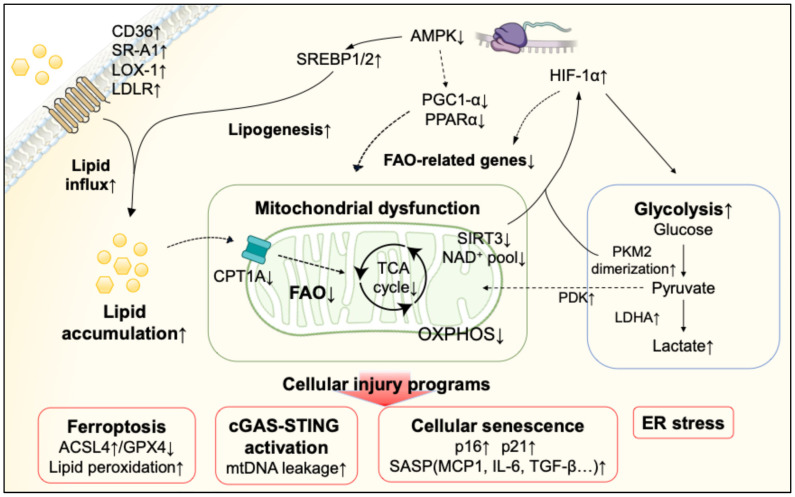
Integrated metabolic and cellular alterations underlying lipotoxic injury in fatty kidney disease. In renal parenchymal cells—most notably proximal tubular cells, podocytes, mesangial cells, and endothelial cells—chronic metabolic stress drives a coordinated rewiring of lipid handling and energy metabolism that converges on tubulointerstitial fibrosis. Lipid influx is augmented by upregulation of the scavenger and lipoprotein receptors CD36, SR-A1, LOX-1, and LDLR, while loss of AMPK activity derepresses SREBP1/2-driven *de novo* lipogenesis. AMPK inactivation concomitantly downregulates PGC-1α and PPARα, leading to transcriptional repression of fatty acid oxidation (FAO)-related genes, including the rate-limiting mitochondrial fatty acid importer CPT1A. Combined with reduced peroxisomal β-oxidation, these changes culminate in intracellular lipid accumulation. The resulting metabolic imbalance precipitates mitochondrial dysfunction, characterized by suppressed FAO, depletion of the NAD^+^ pool, downregulation of SIRT3, attenuated TCA cycle flux, and reduced oxidative phosphorylation (OXPHOS). In parallel, stabilization of HIF-1α drives a maladaptive shift toward aerobic glycolysis: PKM2 dimerization and PDK upregulation divert pyruvate away from mitochondrial oxidation, while elevated LDHA activity sustains lactate production, reinforcing aerobic glycolytic reprogramming that further compromises oxidative metabolism and impairs mitophagy. This metabolic failure activates a network of cellular injury programs (bottom panel) mechanistically implicated in renal fibrogenesis: (i) ferroptosis, marked by ACSL4 upregulation, GPX4 downregulation, and accumulation of lipid peroxides; (ii) cGAS–STING pathway activation triggered by cytosolic leakage of mitochondrial DNA (mtDNA) from damaged mitochondria; (iii) cellular senescence, indicated by induction of p16 and p21 together with a senescence-associated secretory phenotype (SASP) enriched in MCP-1, IL-6, and TGF-β; and (iv) endoplasmic reticulum (ER) stress. Collectively, these injury programs amplify tubular damage, sustain a proinflammatory and profibrotic microenvironment, and drive resident fibroblast and pericyte activation into myofibroblasts, ultimately producing the tubulointerstitial fibrosis that defines fatty kidney disease. Arrows (↑) and (↓) denote upregulation/activation and downregulation/inhibition, respectively. Solid arrows indicate direct activating relationships or metabolic flux, whereas dashed arrows indicate inhibitory or attenuated regulatory relationships. Illustration created using elements from NIAID NIH BIOART Source (bioart.niaid.nih.gov/bioart/352, 422, 523, 651).

Collectively, these findings indicate that mitochondrial dysfunction, metabolic reprogramming, impaired mitochondrial quality control, mtDNA-mediated innate immune activation, and lipid peroxidation form an integrated network that drives renal fibrogenesis (Figure 1).

### 2.4. Senescence, Ferroptosis, and Dysregulated Autophagy

Cellular senescence in the kidney is a critical driver of CKD progression and fibrosis [97]. Multiple murine kidney injury models demonstrated that G2/M-arrested tubular epithelial cells promote renal fibrosis through JNK-dependent profibrotic signaling, whereas inhibition of JNK or p53 attenuated G2/M arrest and ameliorated fibrosis [98]. In naturally aged and irradiation-induced senescent mouse models subjected to ischemia–reperfusion injury, senescent cells promoted renal fibrosis and impaired tubular regenerative capacity, partly through the production of SASP factors including TGF-β1, IL-6, and TNF-α. Furthermore, depletion of senescent cells with the senolytic agent ABT-263 suppressed SASP expression and fibrosis, resulting in improved structural and functional renal recovery after injury [99]. These findings suggest that targeting dysregulated cell cycle control and senescent cells may represent an effective therapeutic strategy to prevent the progression from AKI to CKD.

Autophagy plays a paradoxical role in renal senescence. The proximal tubular S3 segment is the most susceptible to I/R injury and shows rapid autophagy activation after I/R. In GGT-CreERT2/Atg5-flox/flox mice, S3-segment-specific deletion of Atg5 increased early tubular cell death (2 h after I/R injury) but attenuated tubular injury, inflammation, cellular senescence, and fibrosis in the long term [100]. Using an inducible, Pax8-mediated tubule-specific *Atg7* knockout model (Pax8-rtTA/LC1/Atg7^flox/flox^ mice), this study demonstrated that deleting *Atg7* after ischemia–reperfusion injury attenuated chronic tubular injury, senescence, G2/M arrest, and interstitial fibrosis [101]. These results indicate that persistent tubular autophagy drives maladaptive repair and profibrotic transformation. Notably, among several cytokines, only FGF2 was selectively suppressed by autophagy deficiency, suggesting the Atg7–FGF2 axis as a critical paracrine mechanism for fibroblast activation [101]. In addition to this tubule-driven mechanism, recent evidence suggests that the cell cycle regulator Plk1, a key regulator of G2/M progression, may also contribute to fibroblast activation and fibrotic progression through regulation of the autophagy-lysosome axis. Analyses using renal fibroblasts (NRK49F cells) suggested that Plk1 may contribute to the maintenance of lysosomal acidification and autophagic flux through regulation of ATP6V1A phosphorylation. Furthermore, in a UUO model using systemic heterozygous Plk1 knockout mice, Plk1 deficiency was associated with suppression of the autophagy-lysosome axis, accompanied by attenuation of renal interstitial fibrosis and inflammatory cell infiltration [102]. In contrast to the maladaptive effects of persistent autophagy in injured tubular epithelial cells, autophagy in renal fibroblasts appears to exert antifibrotic functions. Recent studies demonstrated that Smoothened (Smo) signaling is upregulated in fibroblasts in multiple CKD models and promotes myofibroblast activation and extracellular matrix production by suppressing autophagic flux through the β-arrestin1/Src/β-catenin/mTOR pathway, whereas genetic deletion or pharmacologic inhibition of Smo restored fibroblast autophagy and attenuated renal fibrosis [103]. Therefore, precise therapeutic strategies that take into account the cell type-specific dynamics of autophagy may be required for the effective control of renal fibrosis.

Beyond cellular senescence, ferroptosis, an iron-dependent form of regulated cell death, has emerged as a potential contributor to maladaptive repair and kidney fibrosis. Acyl-CoA synthetase long-chain family member 4 (ACSL4) promotes the incorporation of polyunsaturated fatty acids into phospholipids, thereby increasing susceptibility to lipid peroxidation and ferroptotic injury [104]. ACSL4 upregulation has been implicated in kidney injury and fibrosis, and genetic or pharmacological inhibition of ACSL4 can attenuate lipid peroxidation, ferroptosis, and renal fibrotic injury in experimental models [105]. Although the direct connection between ACSL4 and mitochondrial energy metabolism remains incompletely defined, ACSL4 may provide a mechanistic link between lipid remodeling, oxidative stress, and fibrotic kidney injury. In a 5/6 nephrectomy-induced CKD rat model, ferroptotic cells were identified in the remnant kidney in association with disordered iron metabolism, characterized by decreased FPN expression and increased NCOA4 expression. Treatment with the iron chelator deferoxamine was associated with reduced TGF-β1/Smad3 signaling activation and attenuation of renal fibrosis [106]. Furthermore, ferroptotic tubular epithelial cells (NRK52E cells) secrete MCP-1, thereby promoting macrophage recruitment and accumulation, which may contribute to interstitial inflammation and subsequent fibrotic progression [107]. Interestingly, the effects of ferroptosis may differ depending on the cell type within the fibrotic kidney. Because TGF-β-activated myofibroblasts exhibit greater sensitivity to ferroptosis than tubular epithelial cells, low-dose administration of the GPX4 inhibitor RSL3 selectively induced myofibroblast ferroptosis and attenuated renal fibrosis in I/R injury mice, albeit accompanied by increased interstitial cell death [108]. Such differential ferroptosis vulnerability may provide a therapeutic window for the selective depletion of profibrotic myofibroblasts while preserving functional renal parenchyma.

Collectively, these findings suggest that senescence, ferroptosis, and dysregulated autophagy constitute interconnected maladaptive repair programs in the injured kidney. Therefore, therapeutic strategies targeting these pathways will likely require precise cell type-specific modulation to suppress fibrosis while preserving adaptive regenerative responses.

### 2.5. Renal–Adipose Crosstalk

Adipose tissue exerts profound regulatory control over renal energy metabolism through secreted adipokines, extracellular vesicles, and anatomically proximate fat depots.

Classical adipokines exert diverse effects on renal structure and function. Leptin, elevated in chronic kidney disease (CKD) as hyperleptinemia, promotes pro-fibrotic responses by functioning as a coactivator of transforming growth factor-β (TGF-β) signaling [109]. Leptin deficiency reduces TGF-β mRNA, Smad2/3 activation, and downstream profibrotic gene induction in a unilateral ureteral obstruction model [109]. In obese mice, leptin treatment of mesangial cells induces thrombospondin-1 (TSP1), TGF-β1, fibronectin, and collagen IV expression [110]. Conversely, adiponectin demonstrates renal-protective properties through activation of AMP-activated protein kinase (AMPK), suppression of oxidative stress, and attenuation of kidney injury [111,112,113]. Adiponectin demonstrates potent renoprotective effects: its receptor agonism activates AMPK and reduces renal injury in contrast-induced nephropathy models [111]; its overexpression attenuates kidney fibrosis in deoxycorticosterone acetate-salt and angiotensin II-induced CKD mice [112]; and it protects renal tubular cells from angiotensin II-induced oxidative stress through AMPK and cAMP-Epac pathways [113]. Adiponectin levels are characteristically reduced in obesity and CKD, contributing to loss of anti-inflammatory and anti-fibrotic protection.

The anatomical relationship between the kidney and adipose tissue assumes particular importance in obesity-related renal disease. Perinephric and renal sinus fat accumulation correlates strongly with metabolic syndrome, hypertension, and chronic kidney disease progression [16,20,114]. In community cohorts, higher renal sinus fat is associated with higher blood pressure and lower estimated glomerular filtration rate [16]. In type 2 diabetes patients, computed tomography-measured perirenal fat thickness independently predicts lower baseline eGFR and higher incidence of CKD after adjustment for visceral adiposity [20], and higher renal sinus fat volume is independently correlated with lower measured GFR, lower effective renal plasma flow, and higher renal vascular resistance [114]. Perinephric adipose tissue undergoes hypoxia in obesity, triggering macrophage infiltration and local inflammatory cytokine production that establishes paracrine gradients affecting adjacent renal parenchyma. The renin-angiotensin system (RAS) represents an additional nexus linking adipose tissue to the kidney. Circulating Angiotensin II (Ang II) levels correlate with body weight, visceral adiposity, and insulin resistance in obese individuals with type 2 diabetes [115]. Human adipose tissue expresses key components of a local renin–angiotensin system, including angiotensinogen, renin, and ACE, supporting local Ang II generation [116], and adipose-specific overexpression of angiotensinogen elevates systemic blood pressure and suppresses renal renin expression through negative feedback [117]. Perirenal fat accumulation is associated with poor antihypertensive response to mineralocorticoid receptor antagonist (MRA) treatment, including smaller reductions in blood pressure and greater initial eGFR decline, in patients with primary aldosteronism. In db/db mice, MRA treatment reduced macrophage infiltration and fibrosis marker expression in perirenal fat [118]. These findings suggest that perirenal fat accumulation may contribute to the association between MRA activation and renal dysfunction in patients with primary aldosteronism. Along with Ang II, leptin, which is elevated in obese patients, further amplifies intrarenal RAS; hyperleptinemia raises plasma Ang II and produces glomerular hypertrophy, inflammation, and proteinuria via AT1 receptor signaling [119].

In addition to adipokine and RAS-mediated signaling, obesity-associated perirenal adipose tissue forms an inflammatory niche containing infiltrating macrophages and senescent cells that secrete TNF-α, IL-6, MCP-1/CCL2, and Oncostatin M (OSM). OSM is an IL-6 family cytokine that is produced in inflammatory settings, promotes fibroblast activation and ECM accumulation, and is deeply involved in fibrosis of various organs [120]. Functional genomics analysis integrating diabetic nephropathy GWAS data with perirenal adipose tissue epigenomic (H3K27ac), transcriptomic, and peak-to-gene regulatory network datasets identified an SVF-specific enhancer harboring the DN-associated variant rs2412980. Regulatory network analysis linked this enhancer to OSM, and enhancer activity was strongly correlated with OSM expression (r = 0.77), suggesting that rs2412980 may influence OSM expression in perirenal adipose tissue. Subsequent transcriptomic and immunostaining analyses showed OSM expression in perirenal adipose tissue and OSMR expression in renal fibroblast-rich compartments, supporting a potential adipose–kidney profibrotic signaling axis mediated through OSM–OSMR signaling. However, direct functional validation of enhancer activity and allele-specific effects remains to be established [121].

Beyond classical endocrine signaling, adipocytes release extracellular vesicles (AdEVs) that directly modulate renal cellular phenotypes. AdEVs isolated from obese individuals promote proinflammatory and profibrotic profiles in human renal tubular epithelial cells and endothelial cells in vitro, inducing expression of inflammatory cytokines (IL-6, IL-1β), extracellular matrix proteins (CTGF, FN1), and altering markers of renal injury (NGAL) and vascular dysfunction (eNOS) [122]. Obesity alters the microRNA (8 upregulated and 75 downregulated, including NF-κB/MAPK- and Wnt-related miRNAs) cargo of adipose-derived stromal cell extracellular vesicles, impairing their capacity to reduce inflammation in injured proximal tubular cells and compromising renal repair mechanisms [123]. These vesicles carry bioactive lipids, microRNAs, and proteins that reprogram recipient cell metabolism, establishing a paracrine mechanism of adipose-kidney communication independent of circulating adipokine concentrations.

Collectively, adipose-kidney crosstalk through endocrine, paracrine, and anatomical mechanisms—mediated by adipokines, extracellular vesicles, ectopic fat depots, and local RAS activation—profoundly disrupts renal energy metabolism and drives tubular dysfunction central to obesity-related kidney disease and diabetic nephropathy.

### 2.6. Cellular Origin of Myofibroblasts in Kidney Fibrosis

Renal fibrosis progresses through the activation and accumulation of myofibroblasts within a proinflammatory microenvironment formed by injured tubular epithelial cells and infiltrating immune cells. Injured tubular epithelial cells release DAMPs and generate reactive oxygen species (ROS), which activate pattern-recognition receptor (PRR)–dependent NF-κB signaling and induce proinflammatory cytokines and chemokines—particularly NF-κB–dependent CCL2 in tubular cells, together with TNF-α and IL-1β from tubular and infiltrating immune cells—thereby promoting recruitment of CCR2^+^ monocytes. These monocyte-derived macrophages further orchestrate the recruitment and activation of additional immune populations, including Th17 cells and neutrophils, and amplify the inflammatory and profibrotic niche through the production of TGF-β1, PDGF, CTGF, and related mediators [124,125]. In vitro, hypoxia-induced renal tubular epithelial cells exhibited impaired PPARα-mediated fatty acid oxidation, activation of the NF-κB/NLRP3 pathway, and increased secretion of IL-18, VEGF, and MCP-1. Conditioned-medium experiments further suggested that these mediators promote activation of interstitial fibroblasts through paracrine signaling [126]. In primary mouse pericytes subjected to hypoxia/reoxygenation, CD36 overexpression increased the expression of PDGFRβ, α-SMA, collagen I, and fibronectin, suggesting the promotion of pericyte-to-myofibroblast transition. Furthermore, CD36 overexpression was associated with mitochondrial dysfunction and enhanced mitochondria-dependent apoptosis [127]. Although lipid metabolic flux was not directly assessed in this study, CD36 is a key lipid-sensing receptor, and these findings raise the possibility that lipid-associated signaling pathways may contribute to pericyte activation and myofibroblast expansion during renal fibrosis.

Traditionally, multiple cellular sources of myofibroblasts have been proposed, including proliferation of resident fibroblasts, epithelial–mesenchymal transition (EMT) [128], endothelial–mesenchymal transition (EndMT) [129], and macrophage-to-myofibroblast transition [130]. However, accumulating evidence from genetic fate-mapping and lineage-tracing studies has suggested that the direct contribution of EMT, EndMT, and MMT to the mature myofibroblast pool may be smaller than previously estimated. Instead, these studies increasingly support interstitial mesenchymal cells—particularly resident fibroblasts and pericytes—as the major source of matrix-producing myofibroblasts in murine experimental renal fibrosis models. Genetic fate-mapping studies have demonstrated that the principal source of myofibroblasts responsible for pathological extracellular matrix deposition in renal fibrosis is interstitial mesenchymal cells derived from FoxD1-positive progenitors [131]. In the unilateral ureteral obstruction (UUO) model in mice, transforming growth factor-β1 (TGF-β1) induces G2/M cell cycle arrest in tubular epithelial cells via upregulation of p21. Concurrently, TGF-β1 activates the JNK pathway, promoting the release of platelet-derived growth factor-B (PDGF-B) as well as TGF-β1 itself. These factors, in turn, stimulate phosphorylation of Smad2 and upregulation of α-smooth muscle actin (α-SMA) in pericytes, thereby driving their transition into myofibroblasts and accelerating fibrotic progression [132]. Furthermore, in mouse models with constitutive activation of PDGFRβ, FoxD1-lineage mesenchymal cells expressing PDGFRβ undergo differentiation and proliferation into myofibroblasts, leading to excessive deposition of pathological extracellular matrix and suggesting that fibrosis per se can directly contribute to renal failure [133]. In addition, recent studies employing dual recombinase-based lineage tracing systems that combine Cre–loxP and Dre–rox technologies (XMTracer) have revealed that the contribution of previously proposed sources—such as tubular epithelial cells, endothelial cells, and macrophages—is limited, at least in the models examined. Instead, the majority of detected myofibroblasts are derived from resident fibroblasts/pericytes [134].

Collectively, current lineage-tracing and single-cell datasets support resident fibroblasts and pericytes as the major direct precursors of renal myofibroblasts in several experimental fibrosis models, although the relative contribution of other cellular sources may vary according to disease context, injury type, and stage of fibrosis. These mesenchymal populations may be activated through paracrine signals released from tubular epithelial cells with impaired FAO.

## 3. Therapeutic Strategies for Fatty Kidney Disease

### 3.1. Established Renoprotective Agents

Fatty kidney disease should be regarded as a disorder of renal lipid handling, mitochondrial dysfunction, and maladaptive metabolic rewiring rather than simply as systemic dyslipidemia. Accordingly, current therapeutic approaches increasingly aim to restore renal metabolic homeostasis in addition to suppressing fibrosis and inflammation.

SGLT2 inhibitors are now firmly established as disease-modifying therapies for CKD. In the DAPA-CKD and EMPA-KIDNEY trials, dapagliflozin and empagliflozin reduced the risk of kidney disease progression or cardiovascular events by 39% and 28%, respectively, across a broad spectrum of CKD, including patients without diabetes [135,136]. Although SGLT2 inhibitors improve glycemic control and body weight, their effects on systemic lipid profiles are modest [137]. Importantly, their efficacy in non-diabetic CKD suggests that mechanisms beyond systemic glucose and lipid metabolism contribute substantially to renoprotection. Experimental studies demonstrate that empagliflozin reduces proximal tubular lipid accumulation through suppression of PPARγ-CD36 signaling [138]. Dapagliflozin increases glucose oxidation and activates AMPK while suppressing mTORC1 signaling in diabetic mice [139]. In addition, dapagliflozin suppresses the HIF-1α-mediated metabolic switch from fatty acid oxidation to glycolysis, thereby ameliorating lipid accumulation and tubulointerstitial fibrosis in streptozotocin-induced diabetic mice [140]. Consistent with these findings, a randomized controlled trial in patients with newly diagnosed type 2 diabetes demonstrated that canagliflozin reduced intrarenal lipid content together with alterations in renal oxygenation assessed by functional MRI [141]. In parallel, single-cell transcriptomic analysis of kidney biopsies from patients with type 2 diabetes demonstrated that SGLT2 inhibitor treatment normalized diabetes-associated metabolic reprogramming in proximal tubular cells, including suppression of glycolytic, gluconeogenic, and tricarboxylic acid cycle pathways together with restoration of mTORC1 signaling toward healthy control levels [142].

GLP-1 receptor agonists have been shown in patients with type 2 diabetes to improve glycemic control, decrease body weight, reduce cardiovascular events, and, for some agents such as semaglutide, slow chronic kidney disease progression. The FLOW trial demonstrated that once-weekly semaglutide significantly reduced the risk of major kidney outcomes, cardiovascular death, and all-cause mortality by 24% in patients with type 2 diabetes and chronic kidney disease [143]. However, mediation analyses of the cardiovascular outcome trials with liraglutide (LEADER) and semaglutide (SUSTAIN 6) suggested that improvements in HbA1c, systolic blood pressure, and body weight accounted for only a portion of the renoprotective effects of GLP-1 receptor agonists, indicating the involvement of additional kidney-protective mechanisms beyond improvements in traditional cardiometabolic risk factors [144]. GLP-1 receptor expression in the kidney appears to be relatively limited, yet the mechanisms underlying the renoprotective effects of GLP-1 receptor agonists remain incompletely understood [145]. Although human data remain limited, in a small exploratory study, liraglutide was associated with reductions in renal triglyceride content and albuminuria [146]. Preclinical studies in diabetic kidney disease mouse models suggest that one potential mechanism involves intrarenal metabolic reprogramming through activation of AMPK signaling. Exendin-4 is taken up by kidney tubular cells via non-selective macropinocytosis, thereby activating the AMPK-mediated fatty acid metabolism pathway, suppressing glycolysis and lipid synthesis, and attenuating lipid peroxidation and ferroptosis in diabetic tubular cells, accompanied by reduced ACSL4 expression [147]. Semaglutide has also been reported to increase β-Klotho, an essential co-receptor for FGF19/21 signaling, and to be associated with AMPK activation, reduced ferroptosis, and improved renal injury [148].

Finerenone, a selective nonsteroidal mineralocorticoid receptor antagonist, has been shown to reduce the risk of kidney disease progression and adverse cardiovascular outcomes in patients with CKD and type 2 diabetes [149,150,151]. More recently, the FIND-CKD trial extended these benefits to non-diabetic CKD, demonstrating a slower annual decline in eGFR and a 23% reduction in the risk of a composite kidney or cardiovascular outcome [152]. Unlike SGLT2 inhibitors and GLP-1 receptor agonists, finerenone exerts minimal effects on glycemic control, body weight, or systemic lipid metabolism. In FIDELIO-DKD, reductions in systolic blood pressure accounted for only 13.8% of the observed kidney benefit [153], suggesting that mechanisms beyond hemodynamic effects contribute substantially to renoprotection. Consistent with this concept, a biomarker substudy of FIGARO-DKD demonstrated modulation of fibrosis-related and metabolic protein signatures during finerenone treatment [154]. However, direct evidence defining the intrarenal mechanisms of finerenone in humans remains limited. Experimental studies suggest that finerenone suppresses inflammation, oxidative stress, and tubular injury through restoration of PI3K/Akt/eNOS signaling and reduction in mitochondrial ROS production [155]. In Western diet-induced kidney disease, finerenone restored ERRγ-dependent mitochondrial homeostasis, improving oxidative phosphorylation, reducing renal cholesterol accumulation, and attenuating innate immune and profibrotic signaling pathways [156].

Thus, among currently available therapies, improvement in ectopic renal lipid accumulation and tubular transcriptional programs has been demonstrated in humans. However, direct evidence for restoration of fatty acid oxidation flux, mitochondrial respiration, mitophagy, or quantitative metabolic flux reprogramming remains unavailable. These observations highlight a persistent gap between mechanistic insights from experimental models and currently measurable metabolic endpoints in clinical studies.

Although direct evidence remains limited, combination therapy may be particularly attractive in fatty kidney disease because currently available agents target distinct but complementary pathways. SGLT2 inhibitors are thought to primarily improve tubular energetics and metabolic flexibility, GLP-1 receptor agonists may modulate nutrient sensing and ferroptosis-related pathways, whereas finerenone appears to suppress mitochondrial stress, inflammation, and fibrosis, at least based on preclinical studies.

Among currently available studies, the most informative clinical data concern combination therapy with an SGLT2 inhibitor and finerenone. In the CONFIDENCE trial, dual treatment with empagliflozin and finerenone reduced UACR by approximately 30% more than either monotherapy at 180 days, consistent with additive effects on kidney injury pathways [157]. Preclinical studies further suggest that finerenone may suppress residual inflammatory and profibrotic pathways that remain active despite RAS/SGLT2 blockade, providing a mechanistic rationale for combination therapy [158].

Evidence for GLP-1 receptor agonist and SGLT2 inhibitor combinations is less developed. In FLOW, the kidney benefits of semaglutide appeared generally preserved among participants receiving background SGLT2 inhibitors, although the trial was not powered to assess additive effects [159]. Consistent with this observation, a large target-trial emulation study reported a lower risk of kidney disease progression following initiation of GLP-1 receptor agonists in patients already receiving SGLT2 inhibitors [160].

However, whether multidrug therapy produces additive improvements in renal lipid handling, mitochondrial function, or metabolic flexibility remains unknown because these endpoints have not been systematically assessed in clinical trials. Future studies integrating kidney outcomes with metabolic phenotyping will be required to determine whether combination therapy can more effectively reverse the metabolic abnormalities underlying fatty kidney disease.

### 3.2. Emerging Therapeutic Targets

Beyond currently approved therapies, emerging interventions for fatty kidney disease increasingly focus on restoring renal metabolic homeostasis in addition to suppressing inflammation and extracellular matrix accumulation. These approaches target multiple aspects of maladaptive metabolic rewiring in tubular cells, including impaired FAO, lipid handling, and mitochondrial dysfunction.

Restoration of FAO remains a central therapeutic concept in kidney disease, and PPARα activation represents one clinically relevant approach to correcting this metabolic defect. Pemafibrate is a selective PPARα modulator (SPPARMα) developed to enhance receptor selectivity while minimizing off-target effects observed with conventional fibrates. In experimental models of fatty acid overload nephropathy, pemafibrate restored renal fatty acid metabolic programs and reduced tubular injury markers [161], supporting the concept that enhancement of FAO may confer renoprotective effects. However, translation of these findings into clinical benefit has proven challenging. In the PROMINENT trial, pemafibrate substantially improved triglyceride-rich lipoprotein profiles but failed to reduce cardiovascular events, and a reversible decline in eGFR was observed during treatment [162]. Despite a strong mechanistic rationale, clinical evidence supporting kidney protection with selective PPARα modulation remains limited. Beyond PPARα activation, several complementary metabolic pathways have emerged as potential therapeutic targets for restoring renal lipid homeostasis. Experimental studies have shown that impaired tubular FAO induces ATP depletion, epithelial dedifferentiation, lipid accumulation, and fibrosis, whereas restoration of CPT1A-mediated mitochondrial FAO attenuates kidney injury [163,164]. Peroxisomal FAO has also emerged as an important contributor to renal metabolic homeostasis, as loss of the rate-limiting peroxisomal β-oxidation enzyme ACOX1 promotes lipid accumulation, ER stress activation, and fibrotic remodeling [84]. In parallel, NAD^+^ replenishment has been identified as a drug-responsive lever to normalize tubular fuel utilization: a recent study demonstrated that nicotinamide riboside increased renal NAD^+^ levels, activated PPARα signaling, restored proximal tubular FAO, and prevented chronic kidney disease progression in Alport syndrome models, with single-nucleus RNA sequencing localizing these effects to proximal tubules [54]. Additional strategies targeting renal lipid accumulation have also been proposed. For example, activation of oxysterol-binding protein-like 7 (OSBPL7) enhanced ABCA1-mediated cholesterol efflux and prevented renal functional decline in experimental models [165].

Mitochondrial quality control, particularly mitophagy, represents a major therapeutic frontier. PHB2 overexpression restores mitochondrial homeostasis and attenuates inflammation, highlighting PHB2-mediated mitophagy as a renoprotective arm of mitochondrial quality control and a potential therapeutic target in CKD [166]. Additional work indicates that targeting nuclear receptors such as ESRRA with mitochondria-directed small molecules can induce ATG5-dependent mitophagy and ameliorate diabetic kidney injury, positioning mitophagy as a mechanistically distinct axis that likely needs to be combined with FAO restoration for durable benefit [167].

In parallel with strategies that restore FAO and mitochondrial quality control, increasing attention has focused on correcting maladaptive metabolic reprogramming characterized by a shift from oxidative metabolism toward glycolysis. PKM2 exists in two functional states: an enzymatically active tetramer that catalyzes phosphoenolpyruvate to pyruvate and a low activity dimer that translocates to the nucleus, where it acts as a coactivator of HIF-1α [168]. TEPP-46 is a small-molecule PKM2 activator that induces PKM2 tetramerization by stabilizing PKM2 subunit interactions [169]. TEPP-46 attenuated renal apoptosis and fibrosis and reduced senescence-associated markers in db/db mice [170]. LDHA inhibition is also a promising therapeutic option. The antiepileptic drug stiripentol inhibits LDHA but is limited by off-target effects, poor aqueous solubility, and instability in acidic environments. In UUO mice, kidney-targeted delivery of stiripentol via a nanoliposomal system reduced lactate accumulation, suppressed TGF-β signaling, and attenuated renal fibrosis [171].

Targeting the TGF-β/Smad axis has long been pursued as a core antifibrotic strategy. However, direct systemic blockade of TGF-β has proven challenging in clinical translation. The phase 2 clinical trial of fresolimumab, a monoclonal anti-TGF-β antibody, for patients with steroid-resistant primary focal segmental glomerulosclerosis did not meet its primary efficacy endpoint of proteinuria reduction [172]. In mouse UUO models, selective downstream inhibition with the specific Smad3 inhibitor SIS3 attenuated fibrosis, inflammation, and apoptosis [173]. Epigenetic and post-transcriptional regulators of the same pathway have begun to emerge as complementary modulators of TGF-β/Smad activity. The kidney-enriched long noncoding RNA lnc-TSI binds to the Mad Homology 2 domain of Smad3 and blocks the TGF-β/Smad3 signaling pathway. Ectopic expression of human lnc-TSI inhibited renal fibrosis and protected renal function in mouse models of both UUO and IRI [174]. MicroRNA-10a/b directly targets TGF-β receptor 1 through a post-transcriptional mechanism, thereby suppressing TGF-β/Smad signaling. Restoration of renal miR-10a/b expression via lentiviral delivery attenuated collagen deposition and podocyte foot process effacement in streptozotocin-induced DKD mice [175].

**Figure 2:**
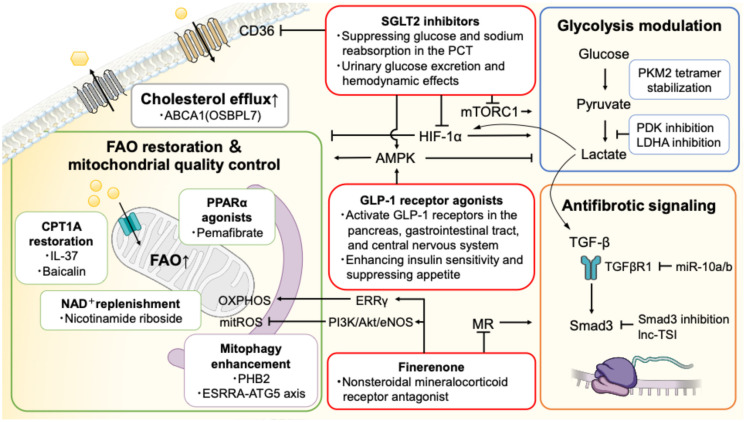
Therapeutic strategies for fatty kidney disease. Pharmacological intervention in fatty kidney disease can be organized along four interconnected axes addressing distinct nodes of the maladaptive metabolic program that drives tubulointerstitial fibrosis. Established renoprotective agents (red boxes) act largely indirectly on renal lipid metabolism, whereas emerging strategies (colored panels) are designed to engage these pathways more directly. (i) FAO restoration and mitochondrial quality control (green panel) is achieved by CPT1A upregulation (IL-37, baicalin), PPARα agonism by pemafibrate, NAD^+^ replenishment with nicotinamide riboside, and mitophagy enhancement via PHB2 and the ESRRA–ATG5 axis. ABCA1-mediated cholesterol efflux through OSBPL7 modulation prevents free-cholesterol accumulation at the plasma membrane. (ii) Glycolysis modulation (blue panel) counters the HIF-1α–driven shift toward aerobic glycolysis through PKM2 tetramer stabilization, PDK inhibition, and LDHA inhibition, thereby restoring pyruvate flux into the TCA cycle and attenuating myofibroblast activation. (iii) Antifibrotic signaling (orange panel) targets the TGF-β/Smad3 axis through Smad3 inhibition and the kidney-enriched long noncoding RNA lnc-TSI, while miR-10a/b post-transcriptionally suppresses TGFβR1 to attenuate collagen deposition and podocyte injury. (iv) Established renoprotective agents (red boxes). SGLT2 inhibitors suppress the PPARγ–CD36 axis and the HIF-1α–driven glycolytic switch; GLP-1 receptor agonists activate AMPK to restore FAO and suppress ACSL4-mediated ferroptosis; finerenone restores PI3K/Akt/eNOS signaling and ERRγ-dependent mitochondrial biogenesis. The renoprotective benefits demonstrated in DAPA-CKD, EMPA-KIDNEY, FLOW, and FIDELITY are mechanistically explained in part by restoration of renal bioenergetic and metabolic homeostasis, with post hoc analyses supporting additive benefit from combination therapy. Arrows (↑/↓) denote up/downregulation; flat-headed lines (⊣) denote inhibition. Red boxes = approved agents; colored panels = mechanistic strategies. Illustration created using elements from NIAID NIH BIOART Source (bio-art.niaid.nih.gov/bioart/352, 422, 439, 523, 651).

These findings support a therapeutic shift from fibrosis-centered approaches toward restoration of adaptive renal metabolic programs through coordinated regulation of FAO, mitochondrial homeostasis, metabolic flexibility, and profibrotic signaling (Figure 2).

## 4. Conclusions

Disordered renal lipid metabolism and pathological lipid accumulation are emerging as upstream drivers of cellular senescence, kidney aging, and tubulointerstitial fibrosis, thereby accelerating chronic kidney disease progression. Across obesity, type 2 diabetes, and aging, increased lipid influx and impaired fatty acid oxidation promote ectopic lipid deposition, mitochondrial dysfunction, ferroptosis, cGAS–STING activation, and profibrotic senescence signaling, ultimately converging on myofibroblast activation. Although SGLT2 inhibitors and GLP-1 receptor agonists confer renoprotection partly by improving metabolic stress and modulating lipid handling, current evidence for direct correction of intrinsic renal lipid metabolic defects in humans remains limited. Therefore, therapeutic strategies specifically aimed at restoring renal lipid homeostasis may provide additional benefit beyond currently available treatments. Thus, therapies that restore fatty acid oxidation, replenish NAD^+^, enhance mitophagy, or suppress ferroptosis may complement current treatments by targeting lipid-driven kidney aging and fibrosis. Renal lipid metabolism, therefore, represents both a central pathogenic hub and a promising therapeutic vulnerability in chronic kidney disease.

## Data Availability

Not applicable.
